# Hyperreactive Onchocerciasis is Characterized by a Combination of Th17-Th2 Immune Responses and Reduced Regulatory T Cells

**DOI:** 10.1371/journal.pntd.0003414

**Published:** 2015-01-08

**Authors:** Gnatoulma Katawa, Laura E. Layland, Alex Y. Debrah, Charlotte von Horn, Linda Batsa, Alexander Kwarteng, Sandra Arriens, David W. Taylor, Sabine Specht, Achim Hoerauf, Tomabu Adjobimey

**Affiliations:** 1 Institute of Medical Microbiology, Immunology and Parasitology (IMMIP), University Hospital Bonn, Bonn, Germany; 2 Advanced School of Medical Biology and Food Technology (ESTBA), University of Lomé, Lomé, Togo; 3 Kumasi Centre for Collaborative Research in Tropical Medicine (KCCR), Kumasi, Ghana; 4 Faculty of Allied Health Sciences and School of Medical Sciences of Kwame Nkrumah University of Science and Technology, Kumasi, Ghana; 5 Institute of Immunology and Infection Research, University of Edinburgh, Edinburgh, Scotland; University of Liverpool, United Kingdom

## Abstract

Clinical manifestations in onchocerciasis range from generalized onchocerciasis (GEO) to the rare but severe hyperreactive (HO)/sowda form. Since disease pathogenesis is associated with host inflammatory reactions, we investigated whether Th17 responses could be related to aggravated pathology in HO. Using flow cytometry, filarial-specific cytokine responses and PCR arrays, we compared the immune cell profiles, including Th subsets, in individuals presenting the two polar forms of infection and endemic normals (EN). In addition to elevated frequencies of memory CD4^+^ T cells, individuals with HO showed accentuated Th17 and Th2 profiles but decreased CD4^+^CD25^hi^Foxp3^+^ regulatory T cells. These profiles included increased IL-17A^+^, IL-4^+^, RORC2^+^ and GATA3^+^CD4^+^ T cell populations. Flow cytometry data was further confirmed using a PCR array since Th17-related genes (IL-17 family members, IL-6, IL-1β and IL-22) and Th2-related (IL-4, IL-13, STAT6) genes were all significantly up-regulated in HO individuals. In addition, stronger *Onchocerca volvulus*-specific Th2 responses, especially IL-13, were observed *in vitro* in hyperreactive individuals when compared to GEO or EN groups. This study provides initial evidence that elevated frequencies of Th17 and Th2 cells form part of the immune network instigating the development of severe onchocerciasis.

## Author Summary

Onchocerciasis, also known as river blindness is a tropical disease causing health and socioeconomic problems in endemic communities especially sub-Saharan Africa. The disease is transmitted by a filarial nematode called *Onchocerca volvulus*, which is spread by the bite of infected *Simulium* black flies. Characteristic disease symptoms include dermatological disorders and eye lesions that can lead to blindness. Two polar forms of clinical manifestations can occur: generalized onchocerciasis (GEO) presenting mild skin disease or the hyperreactive form (HO) exhibiting severe skin disorders and inflammation. The immunological determinants behind such disease polarization are still not fully clarified. Here, we compared the immune profiles of individuals presenting these two polar forms with those of endemic normals (EN): individuals who have no clinical or parasitological evidence of infection despite ongoing exposure to the infectious agent. We could show that HO individuals, in contrast to GEO and EN, simultaneously presented elevated Th17 and Th2 profiles which were accompanied by reduced numbers of Foxp3^+^ regulatory T cells. This study provides initial evidence that elevated frequencies of Th17 and Th2 cells form part of the immune network associated with severe onchocerciasis.

## Introduction

Onchocerciasis is a neglected tropical disease causing both health and socioeconomic problems [1]. Elicited by the parasitic nematode *Onchocerca volvulus (Ov)*, it is transmitted through the bite of infected black flies (genus *Simulium*). Characteristic disease symptoms include dermatological disorders and eye lesions that can lead to blindness [2]. Two polar forms of clinical manifestations can occur: generalized onchocerciasis (GEO) presenting mild skin disease or the hyperreactive form (HO) exhibiting severe skin inflammation (also called sowda if inflammation is unilaterally predominant) [3]–[5]. Although 99% of infected people live in Africa, small pockets of endemicity can be found in Yemen and Central and Southern America [2]. Over 37 million people are currently infected and within those, around 1% develop HO [6].

In endemic areas, putative immune individuals or endemic normals (EN) are persons, who despite permanent exposure to the parasite, remain without infection or clinical signs of disease [4], [5]. Variations in host immune responsiveness include a spectrum of clinical manifestations ranging from i) GEO individuals with high parasite loads, mild pathology but strong regulatory responses to ii) HO individuals presenting few worms but varying degrees of dermal pathology (acute and chronic papular onchodermatitis, leopard skin, and depigmentation); the term “sowda” is reserved for unilateral, extreme hyperreactivity [3]–[6]. *Onchocerca* are long-living and are renowned for modulating host immune regulatory mechanisms [3], [5], [6], [7]. Adult female worms encase themselves in so called onchocercomas in the skin that are composed of various cell types [5], [6], [8], [9]. CD4^+^ T cells have been reported to be the predominant IL-10 secreting cells in onchocerciasis [10]. However, due to the infrequency of HO cases, few studies have addressed the types of cytokine secreting Th subsets or cellular immune profiles in these individuals. Since Th17 cells have been associated with helminth-induced overt pathology [11], [12] we determined here whether they are active in HO individuals. When compared to GEO and EN groups, HO individuals presented elevated Th17 and Th2 profiles which were accompanied by reduced numbers of Foxp3^+^ regulatory T cells (Treg). Upon PCR array analysis, Th17 and Th2-related genes were also up-regulated in HO patients. These data suggest that preventing the development of HO should focus on tipping the Treg/Th17 balance towards a more regulated response.

## Methods

### Study population and ethics

In 2011, adult *O. volvulus-*infected male and female (21–55 years) individuals from an endemic region in Ghana were recruited within the study: "Enhanced Protective Immunity Against Filariasis (EPIAF)", (http://www.filaria.eu/projects/projects/epiaf.html). Ethical clearance was given by the Committee on Human Research Publication and Ethics at the University of Science and Technology in Kumasi, and the Ethics Committee at the University Hospital Bonn. For comparison, samples were collected from 16 mixed gender infection-free volunteers (27–55 years) that had resided in the same area for at least 10 years (EN). These individuals were negative for MF, had no palpable onchocercomas, and had no pathology related to onchocerciasis. Written informed consent was obtained from all individuals.

### Parasitological assessment and antigen preparation

All infected individuals presented at least one nodule and/or skin lesions and were screened for the presence of dermal microfilariae (MF/mg skin) as previously described [13], [14], [15]. Infections with other intestinal helminths (schistosomes, ascaris) and protozoa (*Plasmodium*) were diagnosed using standard methods (Kato-Katz, finger prick and urine analysis) and all individuals donating samples for this study were free of such infections. A soluble antigen extract from *O. volvulus* adult worms (OvAg) was prepared as previously described [15]. In preceding experiments, thawed PBMCs from infected individuals were cultured with the antigen over 7 days to determine the optimal time-point for cytokine measurement (S1A-D Fig.).

### *In vitro* cell culture and cytokine assessment

PBMCs isolation was performed as previously described [15] and followed by cryo-preservation in liquid nitrogen until required [16]. PBMCs were thawed slowly (37°C) and then washed with RPMI 1640 medium supplemented with 10% FCS, gentamycin, penicillin/streptomycin (50 µg/ml) and L-glutamine (292.3 µg/ml), all from PAA (Linz, Austria). In 96-well plates, 1×10^5^ PBMCs/well were left unstimulated or stimulated with OvAg (20 µg/ml) or αCD3/αCD28microbeads (40,000 beads/ml, Dynal/Invitrogen, Carlsbad, USA) in duplicate for 7 days. Cytokine levels were measured from pooled supernatants using a human FlowCytomix Multiplex Th1/Th2/Th9/Th17/Th22 13-plex kit (eBioscience, San Diego, CA, USA). Data were acquired on a FACS Canto flow cytometer (BD Biosciences, Heidelberg, Germany) and analyzed using FlowCytomix Pro3.0 software (eBioscience).

### Flow cytometry

All reagents were obtained from eBioscience and staining was done as previously described [17]. 1×10^5^ cells/100 µl staining buffer were incubated for 30 mins (4°C) with either 1) anti-human CD3-PerCP-Cy5.5, CD16-FITC (clone CB16) and CD56-PE PE (clone CMSSB); 2) anti-human CD4-APC (clone OKT4), anti-CD45RO-FITC (clone UCHL1) and CD45RA-PerCP-Cy5.5 (clone HI100); 3) CD8-APC (clone SK1) and CD14-FITC (clone 61D3) or 4) CD19-APC (clone HIB19) and CD27-FITC (clone LG7F9). For intracellular staining, cells were activated with a Cells Stimulation Cocktail (phorbol 12-myristate 13-acetate {PMA} and Ionomycin) plus Brefeldin A and monensin (eBioscience) for 6 hours. Thereafter, cells were stained with anti-CD4-APC and to assess Treg levels, anti-human CD25-PECy7 (clone BC96) was added as well. After employing Fix-Perm reagent (eBioscience), cells were blocked with normal rat serum and then incubated for 30 mins (4°C) with either 1) anti-human T-bet-PE (clone eBio4B10) and IFN-γ-FITC (clone 4S.B3); 2) GATA3-PE (clone TWAJ) and IL-4-FITC (clone B-S4) 3) RORC2-PE (clone AFKJS-9) and IL-17A-FITC (clone eBio64DEC17) or 4) Foxp3-FITC (clone 236A/E7) and IL-10-PE (clone JES3-9D7). After further washing cells were re-suspended in fix-perm buffer (eBioscience). To correct spectral overlap, fluorescence compensation was done using UltraComp ebeads (eBioscience). Data were acquired and analyzed using a FACS Canto flow cytometer and software (BD Biosciences).

### PCR Array analysis

Gene expression profiles were quantified using the Human Th17 autoimmunity and inflammation PCR array and RT^2^SYBR Green Mastermix kit (Qiagen, Hilden, Germany) according to protocol. In short, PBMCs were stimulated with αCD3/αCD28 microbeads for 3 hours and RNA was extracted using Trizol (Invitrogen). DNA was digested using the DNA-free kit (Invitrogen) and the concentration and the purity of RNA was determined using the NanoDrop 1000 (Peqlab, Erlangen, Germany). Extracted RNA was reverse transcribed using Qiagen Master mix (Qiagen,) and incubated in the Primus Thermocycler (MWG-Biotech, Ebersberg, Germany). Amplification was performed on the RotorGene 6000 (Corbett Research, Sydney, Australia). Data were analyzed by RT^2^ profiler PCR Array data analysis 3.5 software (Qiagen).

### Statistical analysis

Statistical analyses were performed using PRISM 5 programme (GraphPad Software, Inc., La Jolla, USA). Since most of the variables did not show a normal distribution, the following tests were chosen: to compare three groups a Kruskal-Wallis-test was performed and, if significant, followed by a Mann-Whitney–U test for a further comparison of the groups. P-values of 0.05 or less were considered significant.

## Results

### Individuals presenting HO display higher frequencies of monocytes and memory T cells

**Figure 1:**
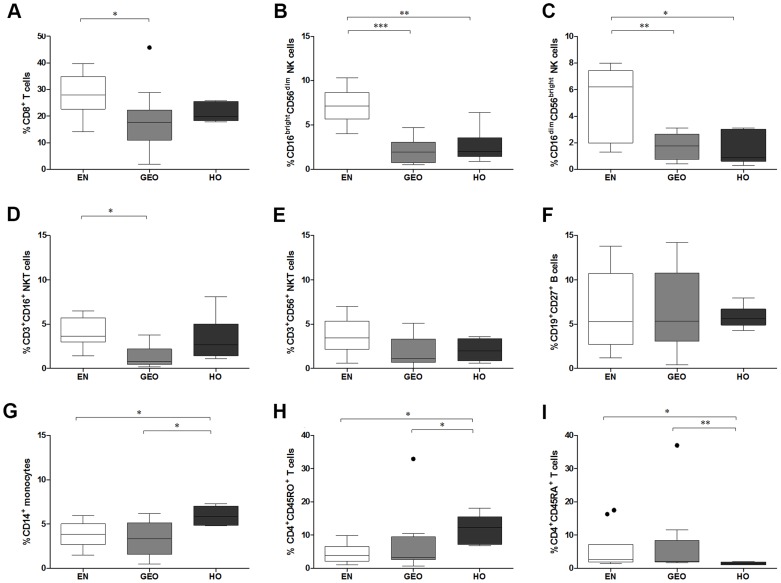
Higher frequencies of monocytes and memory T cells in HO individuals. Isolated PBMCs from EN and individuals presenting either generalized (GEO) or hyperreactive onchocerciasis (HO) were stained with a combination of antibodies to determine the frequencies of CD8^+^ T cells (A); NK cells [CD3^-^CD16^bright^CD56^dim^ (B) and CD3^-^CD16^dim^CD56^bright^ (C)]; NKT cells [CD3^+^CD16^+^ (D) and CD3^+^CD56^+^ (E)]; CD19^+^CD27^+^ memory B cells (F); CD14^+^ monocytes (G); memory CD4^+^ T cells (H) and naive CD4^+^ T cells (I). Graphs show box whiskers (tukey) with outliers from EN n = 10, GEO n = 10 and HO n = 6. Asterisks show statistical differences (Kruskal-Wallis and Mann Whitney test) between the groups indicated by the brackets (*p<0.05, **p<0.01).

To distinguish differences in the immune cell profiles of individuals with onchocerciasis, we analyzed PBMCs for the frequency of innate and adaptive cell populations. Individuals with GEO had significantly less CD8^+^ T cells when compared to EN (Fig. 1A). When compared to levels in EN, both CD16^bright^CD56^dim^ and CD16^dim^CD56^bright^NK populations were significantly lower in GEO and HO individuals but no differences could be observed between infected groups (Fig. 1B and C). CD3^+^CD16^+^ but not CD3^+^CD56^+^NKT cells were also lower in GEO but not HO individuals (Fig. 1D and 1E). Whereas no differences could be observed in the frequency of memory B cells (Fig. 1F), significantly elevated numbers of monocytes (CD14^+^ cells) were identified in HO individuals (Fig. 1G). When compared to GEO and EN groups, HO individuals displayed higher amounts of memory CD4^+^CD45RO^+^ (Fig. 1H) but significantly less naive T cells (Fig. 1I). These data were also reflected when calculated on absolute cell numbers.

### Hyperreactive individuals exhibit dominant IL-4 and IL-17A-producing CD4^+^ T cells

**Figure 2:**
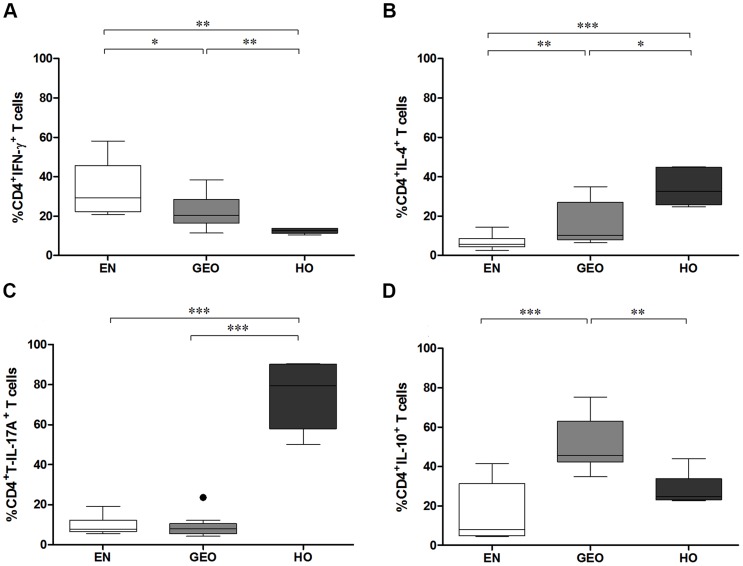
Hyperreactive individuals exhibit a dominant IL-4 and IL-17 phenotype. Isolated PBMCs from EN (n = 10) and *O. volvulus*-infected individuals presenting either GEO (n =  10) or HO (n = 6) were activated with a cell stimulation cocktail for 6 hours. Thereafter, cells were stained with an anti-CD4 antibody and after fixation and permeabilization further stained with anti-human antibodies specific for IFN-γ (A), IL-4 (B), IL-17A (C) and IL-10 (D). Intracellular cytokine expression was determined on the CD4^+^ T cell population by flow cytometry. Graphs show percentages as box whiskers (tukey) with outliers. Asterisks show statistical differences (Kruskal-Wallis and Mann Whitney test) between the groups indicated by the brackets (*p<0.05, **p<0.01, ***p<0.001).

Next, we determined the frequency of cytokine producing CD4^+^ T cells. CD4^+^IFN-γ^+^ T cells were significantly higher in EN when compared to either infected group (Fig. 2A). However, CD4^+^ T cells from GEO individuals did produce more IFN-γ than cells from HO individuals (Fig. 2A). In contrast, infected groups displayed significantly elevated frequencies of IL-4-secreting CD4^+^ T cells when compared to EN groups (Fig. 2B). Moreover, T cells from HO individuals produced more IL-4 than cells from GEO persons (Fig. 2B). Whereas IL-17A-producing CD4^+^ T cells appeared to be a unique characteristic in HO individuals (Fig. 2C), those with GEO had a dominant IL-10 phenotype (Fig. 2D).

### Higher expression of CD4^+^Foxp3^+^ but not CD4^+^CD25^hi^Foxp3^+^ T cells in hyperreactive individuals

**Figure 3:**
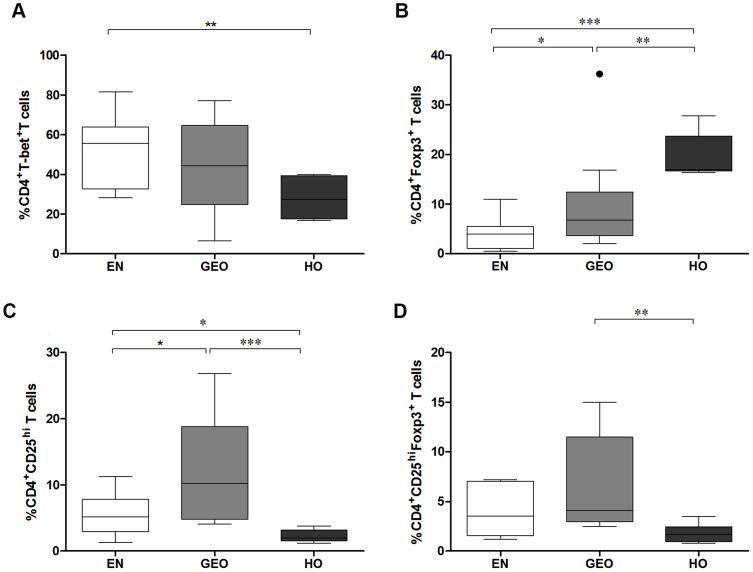
Hyperreactive onchocerciasis individuals present higher frequencies of CD4^+^Foxp3^+^ but not CD4^+^CD25^hi^Foxp3^+^ T cells. PBMCs from EN and *O. volvulus*-infected GEO or HO individuals were activated with a cell stimulation cocktail for 6 hours. Thereafter, cells were stained for CD4 and the transcription factors T-bet (A) or Foxp3 (B). PBMCs fractions were also stained with CD25 to assess the numbers of CD4^+^CD25^hi^ (C) and CD4^+^CD25^hi^Foxp3^+^ (D) T cells in each individual (EN n = 16, GEO n =  16, HO n =  6). Cell population frequencies were determined via flow cytometry. Graphs show percentages as box whiskers (tukey) with outliers. Asterisks show statistical differences (Kruskal-Wallis and Mann Whitney test) between the groups indicated by the brackets (*p<0.05, **p<0.01, ***p<0.001).

In association with their elevated numbers of IFN-γ-producing CD4^+^ T cells (Fig. 2A), EN had significantly higher frequencies of CD4^+^T-bet^+^ T cells when compared to HO individuals (Fig. 3A). Strikingly, the Treg associated transcription factor Foxp3 was strongly expressed by CD4^+^ T cells from hyperreactive individuals, even when compared to GEO individuals (Fig. 3B). Therefore, we expanded our profile panel to include CD25^hi^ expressing CD4^+^ T cells using a previously described protocol and gating strategy [17], [18]. Interestingly, the inclusion of CD25^hi^ cells dramatically changed the profile of CD4^+^ regulatory T cell profile in HO individuals since both CD4^+^CD25^hi^ (Fig. 3C) and CD4^+^CD25^hi^Foxp3^+^ (Fig. 3D) subsets were higher in GEO individuals. This suggests the presence of Foxp3^+^ effector CD4^+^ T cells in persons with HO.

### Th17 and Th2 profiles are enhanced in individuals with hyperreactive onchocerciasis

**Figure 4:**
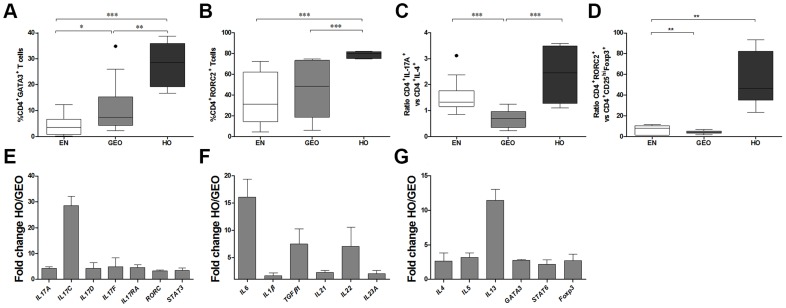
Immune profiles of hyperreactive onchocerciasis individuals are associated with elevated Th2 and Th17 factors. PBMCs isolated from EN (n = 16) and *O. volvulus*-infected GEO (n = 16) or HO (n = 6) individuals were activated with a cell stimulation cocktail for 6 hours. Thereafter, cells were stained for CD4 and the transcription factors GATA3 (A) or RORC2 (B). Ratios of CD4^+^lL-17A^+^ vs. CD4^+^IL-4^+^ T cells (C) and CD4^+^RORC2^+^ vs. CD4^+^CD25^hi^Foxp3^+^ (D) were determined for each individual. Cell population frequencies were determined using flow cytometry. Graphs show percentages as box whiskers (tukey) with outliers. Asterisks show statistical differences (Kruskal-Wallis and Mann Whitney test) between the groups indicated by the brackets (*p<0.05, **p<0.01, ***p<0.001). (E–G) After PBMCs were stimulated with αCD3/αCD28 (40,000 beads/ml) for 3h, RNA was extracted and transcribed into cDNA. Thereafter, PCR Arrays were performed using the RT^2^ Profiler PCR Array Human Th17 kit. Bars show fold change ± SEM increase in the indicated gene expression between four age-matched males presenting either GEO or HO forms of infection.

Correlating with the elevated IL-4 and IL-17 cytokine expression, CD4^+^ T cells from HO individuals had significantly higher levels of both GATA3 (Fig. 4A) and RORC2 (Fig. 4B) transcription factors. In addition, the ratio of CD4^+^IL-17A^+^/CD4^+^IL-4^+^ T cells was higher in individuals with HO (Fig. 4C) when compared to the GEO group. This trend remained when comparing the ratio CD4^+^RORC2^+^/CD4^+^CD25^hi^Foxp3^+^ in HO individuals with both GEO and EN groups (Fig. 4D). Using a Th17-based PCR array, we compared expression levels of Th17, Th2 and Treg related genes in GEO and HO individuals. As shown in Fig. 4E, IL-17 associated genes, such as *IL17A*, *IL17C*, *IL17D, IL17F, RORC* and *STAT3*, were all up-regulated in cells from HO individuals. The genes of cytokines known to be required for the induction of Th17 cells such as IL-6, IL-1β, TGF-β1, IL-21 and IL-23A [19], [20] and the *IL22* gene were also highly up-regulated in HO persons (Fig. 4F). With regards to Th2-related genes and the Treg-associated *foxp3* expression, the *IL13* gene presented the strongest fold increase and correlated to the elevated gene expression of *GATA3* and *STAT6* (Fig. 4G). In correlation with elevated amounts of CD4^+^Foxp3^+^ cells in HO individuals, *foxp3* gene expression in these patients was also upregulated (Fig. 4G).

### Elevated Ov-specific Th2 but not Th17 responses in hyperreactive onchocerciasis individuals

**Figure 5:**
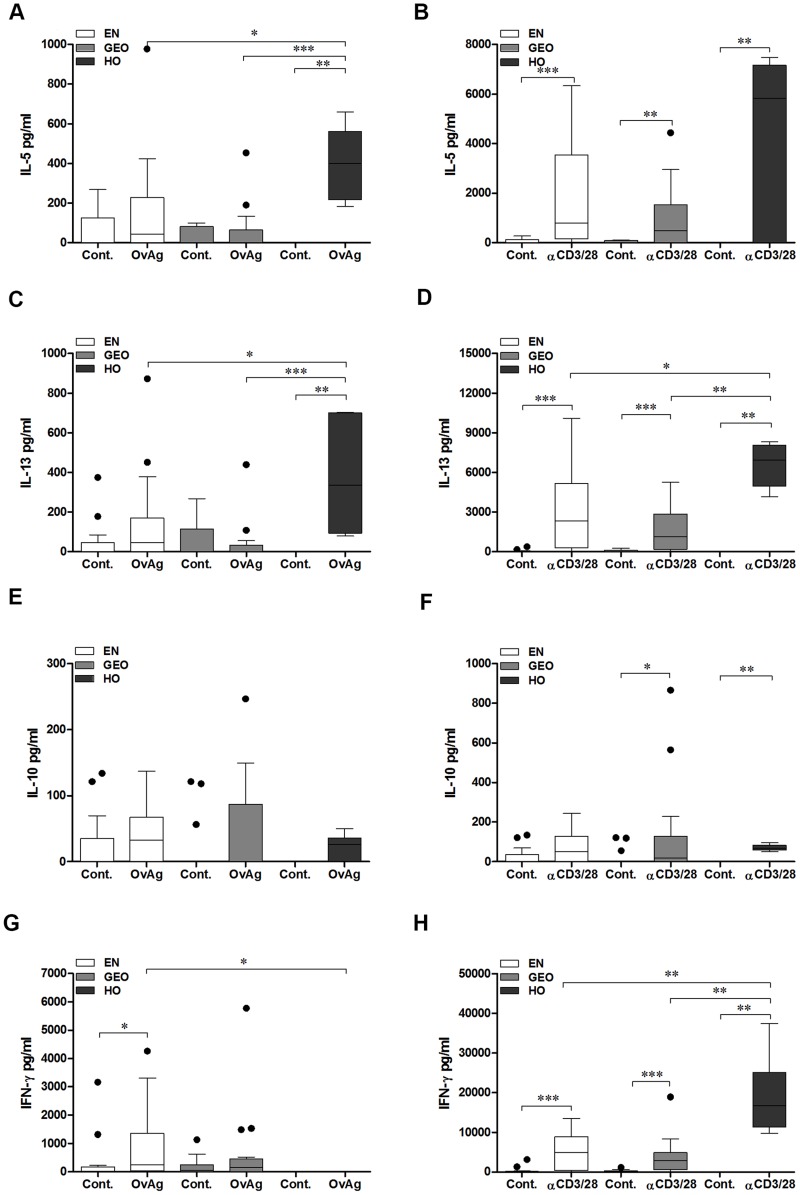
Hyperreactive individuals present stronger filarial-specific Th2 responses *in vitro*. Isolated PBMCs (1×10^5^/well) from EN (n = 16) and *O. volvulus-*infected GEO (n = 16) or HO (n = 6) individuals were left either unstimulated (Cont.) or activated with either *O. volvulus* antigen extract (OvAg, 20 µg/ml) or αCD3/αCD28 (40,000 beads/ml) for 7 days. Thereafter, levels of IL-5 (A, B), IL-13 (C, D), IL-10 (E, F) and IFN-γ (G, H) were measured in the culture supernatants using a FlowCytomix™ Multiplex kit via flow cytometry. Graphs show data as box whiskers (tukey) with outliers. Asterisks show statistical differences (Kruskal-Wallis and Mann Whitney test) between the groups indicated by the brackets (*p<0.05, **p<0.01, ***p<0.001).

**Figure 6:**
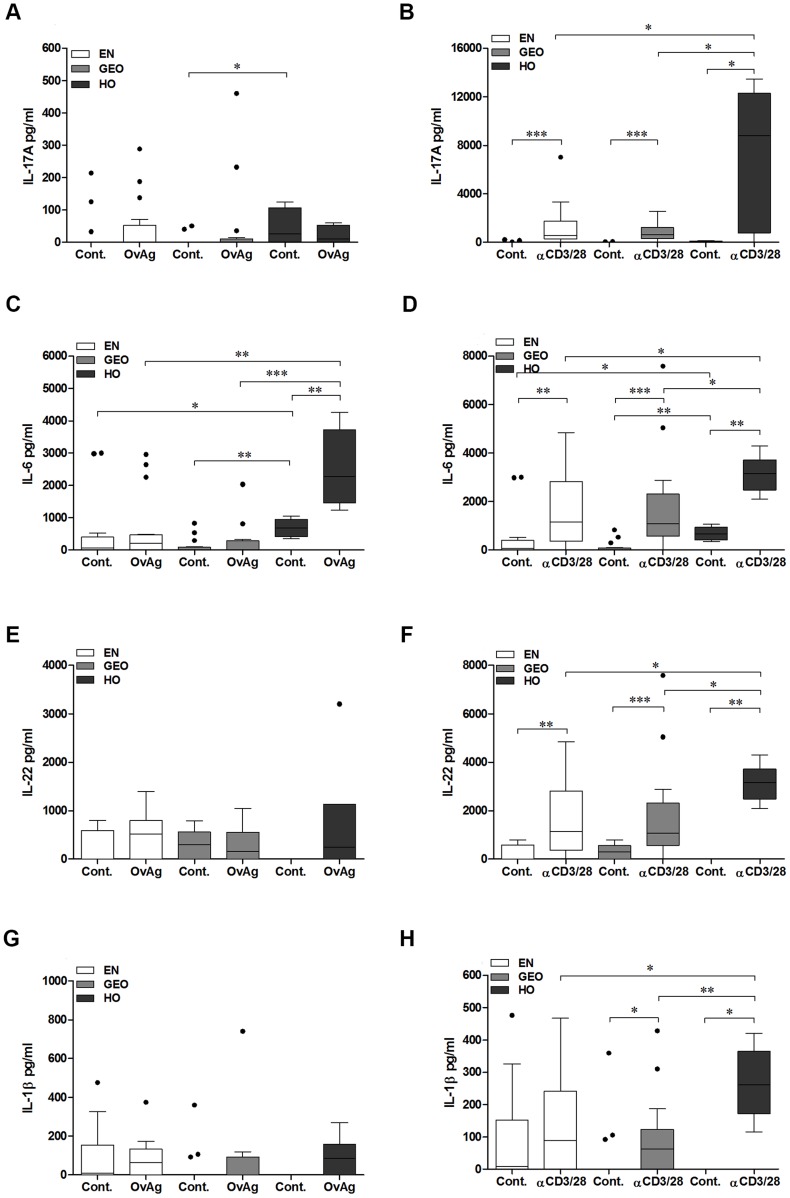
PBMCs from hyperreactive onchocerciasis individuals exhibit strong IL-6 responses following filarial specific activation *in vitro*. Isolated PBMCs (1×10^5^/well) from EN (n = 16) and *O. volvulus*-infected GEO (n = 16) or HO (n = 6) individuals were left either unstimulated (Cont.) or activated with either *O. volvulus* antigen extract (OvAg, 20 µg/ml) or αCD3/αCD28 (40,000 beads/ml) for 7 days. Thereafter, levels of IL-17A (A, B), IL-6 (C, D), IL-22 (E,F) and IL-1β (G,H) were measured in the culture supernatants using a FlowCytomix™ Multiplex kit via flow cytometry. Graphs show data as box whiskers(tukey) with median, interquartile ranges and outliers. Asterisks show statistical differences (Kruskal-Wallis and Mann Whitney test) between the groups indicated by the brackets (*p<0.05, **p<0.01, ***p<0.001).

To measure filarial-specific responses from cyro-preserved PBMCs, cells were cultured with OvAg for 7 days: the optimal time-point for cytokine production (S1A-D Fig.). When compared to EN or GEO groups, PBMCs from HO individuals secreted significantly more IL-5 and IL-13 when activated with either OvAg or αCD3/αCD28 (Fig. 5A-D). Cultures from all groups produced little IL-10 in response to OvAg (Fig. 5E), although infected individuals did produce more IL-10 than control cultures upon activation with αCD3/αCD28 (Fig. 5F). Cultures from EN secreted significantly more IFN-γ upon activation with OvAg (Fig. 5G) which correlates with their CD4^+^ T cell cytokine profile shown in Fig. 2A. The dampened IFN-γ responses from cells of HO individuals was not reflected upon αCD3/αCD28 activation indicating that failure to produce IFN-γ was not a deficit of Th1 cells but dampened filarial-specific IFN-γ-producing cells (Fig. 5H). The induction of Th17 cells requires IL-6, IL-1β, TGF-β and IL-23 [19]. In contrast to the high amounts of IL-17A secreting CD4^+^ T cells observed by flow cytometry (Fig. 2C), upon culturing with OvAg, only low levels of IL-17A were detected in the culture supernatants in the HO group. Nevertheless, the basal levels of IL-17A were significantly higher than the basal levels in culture supernatants from the GEO group (Fig. 6A). Upon activation with αCD3/αCD28 however, cells from HO individuals presented significantly higher levels of IL-17A when compared to both EN and GEO group (Fig. 6B), reflecting again the findings via flow cytometry (Fig. 2C). IL-6 levels from cultures of PBMCs from HO individuals were significantly higher than the other groups (Fig. 6C and D). As with IL-17A, no significant differences in the levels of IL-22 or IL-1β following stimulation with OvAg were observed (Fig. 6E and G respectively). However, after activation with αCD3/αCD28, PBMCs cultures from HO individuals secreted elevated amounts of IL-22 and IL-1β when compared to EN and GEO groups (Fig. 6F and H respectively). Thus, although the overall filarial-specific Th17-related responses were not highly significant in hyperreactive individuals, these cytokines were enhanced upon αCD3/αCD28 activation indicating a biased Th17 inflammatory profile.

## Discussion

Cases of HO are infrequent (probably 1% of the infected population) and the underlying etiology has only partially been elucidated [4], [5], [6]. After comparing cellular immune profiles we have determined that an accentuated Th17/Th2 phenotype forms part of the immune network which drives the development of hyperreactive onchocerciasis. Th1 cells do not contribute to this stage of pathogenesis since T-bet^+^ and IFN-γ producing CD4^+^ T cells in HO individuals were significantly lower when compared to EN. Indeed, both CD4^+^IFN-γ^+^ T cells and IFN-γ levels released following OvAg stimulation by PBMCs were significantly higher in the EN group. The association of IFN-γ and putative immunity in endemic-residing individuals has been demonstrated in studies investigating reactions to L3 larvae [21]. Cooper et al. further demonstrated that early exposure to infection elicited elevated IFN-γ responses to OvAg but not L3 larvae [22]. Since recent reports have suggested that *Wolbachia*, the endosymbiotic bacteria in *O. volvulus*, are the principal activator of innate and Th1 inflammatory immunity [4], these responses may stem from exposure to worms and/or bacteria.

Previous studies have noted that HO individuals present elevated numbers of peripheral leucocytes including eosinophils but not neutrophils [23], [24]. Brattig et al. [23] also found no differences in CD19^+^ B cells and expanding on those findings we observed no alterations in memory B cells either. Interestingly, GEO individuals presented reduced numbers of CD8^+^ T cells, NK and NKT cells. Since these individuals have higher worm burdens there is likely elevated amounts of helminth-derived glycolipids and glycoproteins, which may lead to migration of NK and NKT cells into the skin, resulting in decreased numbers in blood. In contrast to *O. volvulus-*infected individuals, EN presented elevated CD16^bright^CD56^dim^ and CD16^dim^CD56^bright^ NK cells. The role of NK cells during filariasis is not well defined although studies with the murine model, *Litomosoides sigmodontis*, showed that depletion of NK cells enhanced worm load and Th2 responses [25]. Previous *in vitro* investigations using PBMCs from healthy individuals demonstrated that NK activation and consequential apoptosis resulted from contact with IL-12-producing monocytes after stimulation with filarial antigens [26]. Therefore, the observed increase of monocytes in HO individuals could be initiated by a) an elevated requirement of phagocytosis due to increased apoptotic material, b) increased stimulation due to dying or dead filariae or c) simply elevated APC requirement due to hyperreactivity.

An increased requirement for antigen presentation in HO individuals would correlate with their elevated levels of memory CD4^+^ T cells, an immunological difference not previously reported between the two polar versions of *O. volvulus* infection. In association, CD45RO^+^ cells have been observed in nodules from sowda individuals via immunohistochemistry [27]. Alongside the immunohistochemical observations of Foxp3^+^ and TGF-β^+^ cells in nodules of GEO but not HO individuals [8], [9], our current data substantiates the known regulatory phenotype in GEO persons. Indeed, we show that GEO individuals have higher frequencies of CD4^+^IL-10-producing T cells which further correlates with studies demonstrating IL-10-secreting Tr1 cells cloned from tissue surrounding the onchocercomas [28]. Moreover, CD4^+^ T cells have been shown to be the largest producers of IL-10 in *O. volvulus*-infected individuals via flow cytometry and although nearly a fifth of those cells further secreted IL-4 hardly any produced IFN-γ [10]. It will be interesting to investigate multifunctional Th17 cells in onchocerciasis, especially HO individuals. Surprisingly, the number of CD4^+^Foxp3^+^ T cells were elevated in HO individuals and following PCR array analysis we also observed higher gene expression levels of *foxp3* in this group without mitogen stimulation. However, upon analysis of the classical Treg phenotype (CD4^+^CD25^hi^Foxp3^+^), we confirmed that GEO patients have higher numbers of this regulatory T cell population. Although mitogen stimulation during the flow cytometry process could have boasted Foxp3 levels in T cells *per se*, future studies will be required to investigate whether these CD4^+^Foxp3^+^ T cells in the HO individuals have any functional relevance or simply reflects the hyperresponsive profile in these patients [5], [29].

A major new finding in this present study is the dominant Th17/Th2 phenotype in HO individuals. Indeed, comparing the cytokine profiles of activated PBMCs from infected individuals revealed that HO patients secreted higher amounts of IL-5 and IL-13 but not IL-10. Such observations were further confirmed via PCR arrays since Th2 genes, especially IL-13, were up-regulated in HO individuals. This correlates to studies showing an increased likelihood for developing sowda in persons carrying the Arg110 variant of IL-13 which leads to higher IL-13 signalling [30]. Th2 responses in filarial infections are linked to infection resistance [31], [32] and microfilariae can elicit pro-inflammatory responses when they are degenerated or moribund [33]. Thus, a potential scenario for developing hyperresponsiveness may be that deviated Th2 responses provoke microfilariae death, which in turn induces a Th17 phenotype. It will be interesting to investigate in the future whether such elevated Th17 responses are induced by microfilariae-derived antigen preparations or recombinant microfilarial proteins. In the study performed here, CD4^+^IL-17A-secreting T cells were 4 times higher in HO individuals and this dominant Th17 phenotype was further confirmed by the higher expression of RORC2 at both the protein and mRNA level. Interestingly, the pronounced IL-17A phenotype was further observed upon TCR activation and the basal level of this cytokine was significantly higher in culture supernatants from PBMCs of HO individuals when compared to the GEO group. Th17 cells are promoted by the inhibition of Foxp3 by IL-6 and elevated TGF-β and IL-1β responses. IL-1β, especially in synergy with IL-23, plays an essential role in the induction and expansion of Th17 cells [34]. In the PCR array, all essential Th17-related genes were up-regulated in HO individuals including IL-22, IL-23A, IL-21, RORC2 and STAT3 genes. Although other genes such as IL-6 and IL-1β were also up-regulated in HO individuals, further investigations would be required to ascertain whether they have other functions than just the induction of Th17 responses. From the 84 analysed genes, 16 were down-regulated in the HO patients and included CCR4, TLR4, the IL-7R and members of the IL-12 family. Interestingly, since environments which enhance IL-7/IL-7R signalling favour alloreactive and autoreactive T cells expansion due to Treg inhibition [35], this would fit to the diminished Treg (CD4^+^CD25^hi^Foxp3^+^) numbers in the HO group.

A protective role of Th2 cytokines elicited during helminth infection, especially in regards to mediating milder forms of pathology, is well established [36]. Recent studies have reported relevant associations between pathology and Th17 characteristics [37]. For example, IL-17A producing cells have been shown to play a significant role in allergic rhinitis [38], allergic contact dermatitis [39] and other immunoinflammatory disorders including psoriatic arthritis, multiple sclerosis and asthma [37], [40], [41]. Indeed, with regards to the latter, strong Th17/Th2 immune responses during allergic asthma result in different clinical manifestations [41]. The relationship between pathology and Th17 cells has been extensively studied in murine schistosome models and revealed that Th17 cells instigated the development of aggravated egg-induced pathology in schistosomiasis [42]. Indeed, the more pronounced granulomatous inflammation in *Schistosome japonicum* infections, was ameliorated upon neutralization of IL-17 *in vivo* [43]. Interestingly, co-infection of *S. mansoni* with the nematode *Heligmosomoides polygyrus* in CBA mice, that develop severe immunopathology, reduced granuloma development and diverted the dominant IL-17 and IFN-γ granuloma-secreting phenotype into one producing Th2-related cytokines instead [44]. The mechanisms behind this modulatory capacity of *H. polygyrus* requires further investigation but it has been suggested that it might lie in changes to the gut microbiota [45]. In lymphatic filariasis (LF), increased Th17 responses have been observed in individuals with chronic lymphoedema and are prominent in patients who have cleared bloodstream microfilariae [38]. This raises the question therefore whether microfilariae normally down-regulate Th17 responses to extend their survival. A recent study on *Schistosoma haematobium*-infected individuals has also revealed an association between Th17 responses and enhanced pathology [12]. Nevertheless, despite the association of pathology and Th17 cells our findings in *O. volvulus*-infected individuals differ from those studies in two regards: 1) Patients presenting filarial lymphoedema, had elevated Th1 and Th17 *but not* Th2 responses following filarial-specific re-stimulation and had no alterations in the amount of secreted IL-10 either [11]. 2) In the study using *S. haematobium-*infected children [12], Treg frequencies were equal amongst the infected and control groups whereas in the HO cohort studied here, individuals had reduced numbers of IL-10^+^CD4^+^ and CD25^hi^Foxp3^+^CD4^+^ Treg when compared to the GEO group. Indeed when investigating the Th17/Treg balance, the ratio of CD4^+^IL-17A^+^/CD4^+^IL-10^+^ and CD4^+^RORC2/CD4^+^CD25^hi^Foxp3^+^ was higher in the HO group suggesting prominent Th17 responses in HO persons and dominant Treg in GEO individuals. Thus, although Th17 cells appear to be a common denominator in helminth-infected individuals displaying severe pathology, each type of infection appears to have created its own subtle collaboration of immune parameters such as Treg or IL-10. Indeed, we show here that the Th17 milieu in individuals with HO is uniquely linked to elevated Th2 responses as well.

## Supporting Information

S1 Fig **Optimal time point for collecting cell culture supernatants.** Thawed PBMCs (1×10^5^/well) from *Onchocerca volvulus*-infection free individuals (n = 4) were left alone (Cont.) or stimulated with either *O. volvulus* antigen extract (20 µg/ml) or αCD3/αCD28 (40,000 beads/ml). Supernatants were collected on day 1 (d1), 3 (d3) and 7(d7). Secretion levels of IFN-γ (A), IL-10 (B), IL-5 (C), and IL-17A (D) were then measured by ELISA. Bars represent mean ± SD of cytokines levels.
(TIF)

## References

[1] Crump A, Morel CM, Omura S (2012) The onchocerciasis chronicle: from the beginning to the end? Trends Parasitol 28: 280–288. 22633470 10.1016/j.pt.2012.04.005

[2] Progress toward elimination of onchocerciasis in the Americas - 1993–2012. MMWR Morb Mortal Wkly Rep 62: 405–408. PMC4604938 23698606

[3] Taylor MJ, Hoerauf A, Bockarie M (2010) Lymphatic filariasis and onchocerciasis. Lancet 376: 1175–1185. 20739055 10.1016/S0140-6736(10)60586-7

[4] Tamarozzi F, Halliday A, Gentil K, Hoerauf A, Pearlman E, et al (2011) Onchocerciasis: the role of Wolbachia bacterial endosymbionts in parasite biology, disease pathogenesis, and treatment. Clin Microbiol Rev 24: 459–468. 21734243 10.1128/CMR.00057-10 PMC3131055

[5] Adjobimey T, Hoerauf A (2010) Induction of immunoglobulin G4 in human filariasis: an indicator ofimmunoregulation. Ann Trop Med Parasitol 104: 455–464. 20863434 10.1179/136485910X12786389891407 PMC3065634

[6] Brattig NW (2004) Pathogenesis and host responses in human onchocerciasis: impact of Onchocerca filariae and Wolbachia endobacteria. Microb Infect 6: 113–128. 10.1016/j.micinf.2003.11.003 14738900

[7] Subrahmanyam D, Mehta K, Nelson DS, Rao YV, Rao CK (1978) Immune reactions in human filariasis. J Clin Microbiol 8: 228–232. 359589 10.1128/jcm.8.2.228-232.1978 PMC275191

[8] Korten S, Kaifi JT, Buttner DW, Hoerauf A (2010) Transforming growth factor-beta expression by host cells is elicited locally by the filarial nematode Onchocerca volvulus in hyporeactive patients independently from Wolbachia. Microb Infect 12: 555–564. 10.1016/j.micinf.2010.03.011 20359544

[9] Korten S, Badusche M, Buttner DW, Hoerauf A, Brattig N, et al (2008) Natural death of adult *Onchocerca volvulus* and filaricidal effects of doxycycline induce local FOXP3+/CD4+ regulatory T cells and granzyme expression. Microb Infect 10: 313–324. 10.1016/j.micinf.2007.12.004 18339571

[10] Mitre E, Chien D, Nutman TB (2008) CD4+ (and Not CD25+) T Cells Are the Predominant Interleukin-10-Producing Cells in the Circulation of Filaria-Infected Patients. J Infect Dis 197: 94–101. 18171291 10.1086/524301

[11] Babu S, Bhat SQ, Pavan Kumar N, Lipira AB, Kumar S, et al (2009) Filarial lymphedema is characterized by antigen-specific Th1 and th17 proinflammatory responses and a lack of regulatory T cells. PLoS Negl Trop Dis 3: e420. 19381284 10.1371/journal.pntd.0000420 PMC2666805

[12] Mbow M, Larkin BM, Meurs L, Wammes LJ, de Jong SE, et al (2013) T-helper 17 cells are associated with pathology in human schistosomiasis. J Infect Dis 207: 186–195. 23087431 10.1093/infdis/jis654 PMC3571236

[13] Mand S, Marfo-Debrekyei Y, Debrah A, Buettner M, Batsa L, et al (2005) Frequent detection of worm movements in onchocercal nodules by ultrasonography. Filaria J 4: 1. 15788103 10.1186/1475-2883-4-1 PMC1079913

[14] Hoerauf A, Specht S, Buttner M, Pfarr K, Mand S, et al (2008) Wolbachia endobacteria depletion by doxycycline as antifilarial therapy has macrofilaricidal activity in onchocerciasis: a randomized placebo-controlled study. Med Microbiol Immunol 197: 295–311. 17999080 10.1007/s00430-007-0062-1 PMC2668626

[15] Arndts K, Specht S, Debrah AY, Tamarozzi F, Klarmann Schulz U, et al (2014) Immunoepidemiological profiling of onchocerciasis patients reveals associations with microfilaria loads and ivermectin intake on both individual and community levels. PLoS Negl Trop Dis 8: e2679. 24587458 10.1371/journal.pntd.0002679 PMC3930501

[16] Satoguina J, Mempel M, Larbi J, Badusche M, Löliger C, et al (2002) Antigen-specific T regulatory-1 cells are associated with immunosuppression in a chronic helminth infection (onchocerciasis). Microb Infect 4: 1291–1300. 10.1016/s1286-4579(02)00014-x 12443893

[17] Adjobimey T, Satoguina J, Oldenburg J, Hoerauf A, Layland LE (2014) Co-activation through TLR4 and TLR9 but not TLR2 skews Treg-mediated modulation of Igs and induces IL-17 secretion in Treg: B cell co-cultures. Innate Immunol 20: 12–23. 10.1177/1753425913479414 23529856

[18] Satoguina JS, Adjobimey T, Arndts K, Hoch J, Oldenburg J, et al (2008) Tr1 and naturally occurring regulatory T cells induce IgG4 in B cells through GITR/GITR-L interaction, IL-10 and TGF-beta. Eur J Immunol 38: 3101–3113. 18924213 10.1002/eji.200838193

[19] Yosef N, Shalek AK, Gaublomme JT, Jin H, Lee Y, et al (2013) Dynamic regulatory network controlling TH17 cell differentiation. Nature 496: 461–468. 23467089 10.1038/nature11981 PMC3637864

[20] Stockinger B, Veldhoen M (2007) Differentiation and function of Th17 T cells. Curr Opin Immunol 19: 281–286. 17433650 10.1016/j.coi.2007.04.005

[21] Turaga PS, Tierney TJ, Bennett KE, McCarthy MC, Simonek SC, et al (2000) Immunity to onchocerciasis: cells from putatively immune individuals produce enhanced levels of interleukin-5, gamma interferon, and granulocyte-macrophage colony-stimulating factor in response to *Onchocerca volvulus* larval and male worm antigens. Infect Immunol 68: 1905–1911. 10722581 10.1128/iai.68.4.1905-1911.2000 PMC97365

[22] Cooper PJ, Mancero T, Espinel M, Sandoval C, Lovato R, et al (2001) Early Human Infection with *Onchocerca volvulus* Is Associated with an Enhanced Parasite-Specific Cellular Immune Response. J Infect Dis 183: 1662–1668. 11343216 10.1086/320709

[23] Brattig NW, Tischendorf FW, Albiez EJ, Buttner DW, Berger J (1987) Distribution pattern of peripheral lymphocyte subsets in localized and generalized form of onchocerciasis. Clin Immunol Immunopathol 44: 149–159. 3608247 10.1016/0090-1229(87)90062-6

[24] Rubio de Krömer MT, Krömer M, Lüersen K, Brattig NW (1998) Detection of a chemotactic factor for neutrophils in extracts of female Onchocerca volvulus. Acta Trop 71: 45–56. 9776142 10.1016/s0001-706x(98)00044-8

[25] Korten S, Volkmann L, Saeftel M, Fischer K, Taniguchi M, et al (2002) Expansion of NK cells with reduction of their inhibitory Ly-49A, Ly-49C, and Ly-49G2 receptor-expressing subsets in a murine helminth infection: contribution to parasite control. J Immunol 168: 5199–5206. 11994476 10.4049/jimmunol.168.10.5199

[26] Babu S, Blauvelt CP, Nutman TB (2007) Filarial parasites induce NK cell activation, type 1 and type 2 cytokine secretion, and subsequent apoptotic cell death. J Immunol 179: 2445–2456. 17675506 10.4049/jimmunol.179.4.2445

[27] Brattig NW, Tenner-Racz K, Korten S, Hoerauf A, Buttner DW (2010) Immunohistology of ectopic secondary lymph follicles in subcutaneous nodules from patients with hyperreactive onchocerciasis (sowda). Parasitol Res 107: 657–666. 20524133 10.1007/s00436-010-1912-0 PMC2919851

[28] Doetze A, Satoguina J, Burchard G, Rau T, Loliger C, et al (2000) Antigen-specific cellular hyporesponsiveness in a chronic human helminth infection is mediated by T(h)3/T(r)1-type cytokines IL-10 and transforming growth factor-beta but not by a T(h)1 to T(h)2 shift. Int Immunol 12: 623–630. 10784608 10.1093/intimm/12.5.623

[29] Magg T, Mannert J, Ellwart JW, Schmid I, Albert MH (2012) Subcellular localization of FOXP3 in human regulatory and nonregulatory T cells. Eur J Immunol 42: 1627-1638.30. Hoerauf A, Kruse S, Brattig NW, Heinzmann A, Mueller-Myhsok B, et al. (2002) The variant Arg110Gln of human IL-13 is associated with an immunologically hyper-reactive form of onchocerciasis (sowda). Microb Infect 4: 37–42. 10.1016/s1286-4579(01)01507-6 11825773

[30] Hoerauf A, Kruse S, Brattig NW, Heinzmann A, Mueller-Myhsok B, et al (2002) The variant Arg110Gln of human IL-13 is associated with an immunologically hyper-reactive form of onchocerciasis (sowda). Microb Infect 4: 37–42. 10.1016/s1286-4579(01)01507-6 11825773

[31] Hoerauf A, Brattig N (2002) Resistance and susceptibility in human onchocerciasis – beyond Th1 vs Th2. Trend Parasitol 18: 25–31. 10.1016/s1471-4922(01)02173-0 11850011

[32] Kerepesi LA, Leon O, Lustigman S, Abraham D (2005) Protective Immunity to the Larval Stages of Onchocerca volvulus Is Dependent on Toll-Like Receptor 4. Infect Immunol 73: 8291–8297. 16299326 10.1128/IAI.73.12.8291-8297.2005 PMC1307100

[33] Brattig NW, Bazzocchi C, Kirschning CJ, Reiling N, Büttner DW, et al (2004) The Major Surface Protein of Wolbachia Endosymbionts in Filarial Nematodes Elicits Immune Responses through TLR2 and TLR4. J Immunol 173: 437–445. 15210803 10.4049/jimmunol.173.1.437

[34] Manel N, Unutmaz D, Littman DR (2008) The differentiation of human TH-17 cells requires transforming growth factor-[beta] and induction of the nuclear receptor ROR[gamma]t. Nat Immunol 9: 641–649. 18454151 10.1038/ni.1610 PMC2597394

[35] Heninger AK, Theil A, Wilhelm C, Petzold C, Huebel N, et al (2012) IL-7 abrogates suppressive activity of human CD4+CD25+FOXP3+ regulatory T cells and allows expansion of alloreactive and autoreactive T cells. J Immunol 189: 5649–5658. 23129754 10.4049/jimmunol.1201286

[36] Allen JE, Maizels RM (2011) Diversity and dialogue in immunity to helminths. Nat Rev Immunol 11: 375–388. 21610741 10.1038/nri2992

[37] van den Berg WB, McInnes IB (2013) Th17 cells and IL-17 a—focus on immunopathogenesis and immunotherapeutics. Semin Arthritis Rheum 43: 158–170. 24157091 10.1016/j.semarthrit.2013.04.006

[38] Tsvetkova-Vicheva V, Konova E, Lukanov T, Gecheva S, Velkova A, et al (2014) Interleukin-17 producing T cells could be a marker for patients with allergic rhinitis. Isr Med Assoc J 16: 358–362. 25058997

[39] Peiser M. Role of Th17 cells in skin inflammation of allergic contact dermatitis. Clin Dev Immunol. 2013: 261037. 10.1155/2013/261037 PMC3759281 24023564

[40] Lynde CW, Poulin Y, Vender R, Bourcier M, Khalil S (2014) Interleukin 17A: toward a new understanding of psoriasis pathogenesis. J Am Acad Dermatol 71: 141–150. 24655820 10.1016/j.jaad.2013.12.036

[41] Cosmi L, Liotta F, Maggi E, Romagnani S, Annunziato F (2011) Th17 cells: new players in asthma pathogenesis. Allergy 66: 989–998. 21375540 10.1111/j.1398-9995.2011.02576.x

[42] Larkin BM, Smith PM, Ponichtera HE, Shainheit MG, Rutitzky LI, et al (2012) Induction and regulation of pathogenic Th17 cell responses in schistosomiasis. Semin Immunopathol 34: 873–888. 23096253 10.1007/s00281-012-0341-9 PMC3690599

[43] Zhang Y, Chen L, Gao W, Hou X, Gu Y, et al (2012) IL-17 neutralization significantly ameliorates hepatic granulomatous inflammation and liver damage in Schistosoma japonicum infected mice. Eur J Immunol 42: 1523–1535. 22678906 10.1002/eji.201141933

[44] Bazzone LE, Smith PM, Rutitzky LI, Shainheit MG, Urban JF, et al (2008) Coinfection with the intestinal nematode Heligmosomoides polygyrus markedly reduces hepatic egg-induced immunopathology and proinflammatory cytokines in mouse models of severe schistosomiasis. Infect Immun 76: 5164–5172. 18710859 10.1128/IAI.00673-08 PMC2573333

[45] Walk ST, Blum AM, Ewing SA, Weinstock JV, Young VB (2010) Alteration of the murine gut microbiota during infection with the parasitic helminth Heligmosomoides polygyrus. Inflamm Bowel Dis 16: 1841–1849. 20848461 10.1002/ibd.21299 PMC2959136

