# Supplementary figures and images for: Hyperreactive Onchocerciasis is Characterized by a Combination of Th17-Th2 Immune Responses and Reduced Regulatory T Cells
Source: PLoS Negl Trop Dis. 2015 Jan 8;9(1):e3414. doi: 10.1371/journal.pntd.0003414 (PMC4288720; doi:10.1371/journal.pntd.0003414)

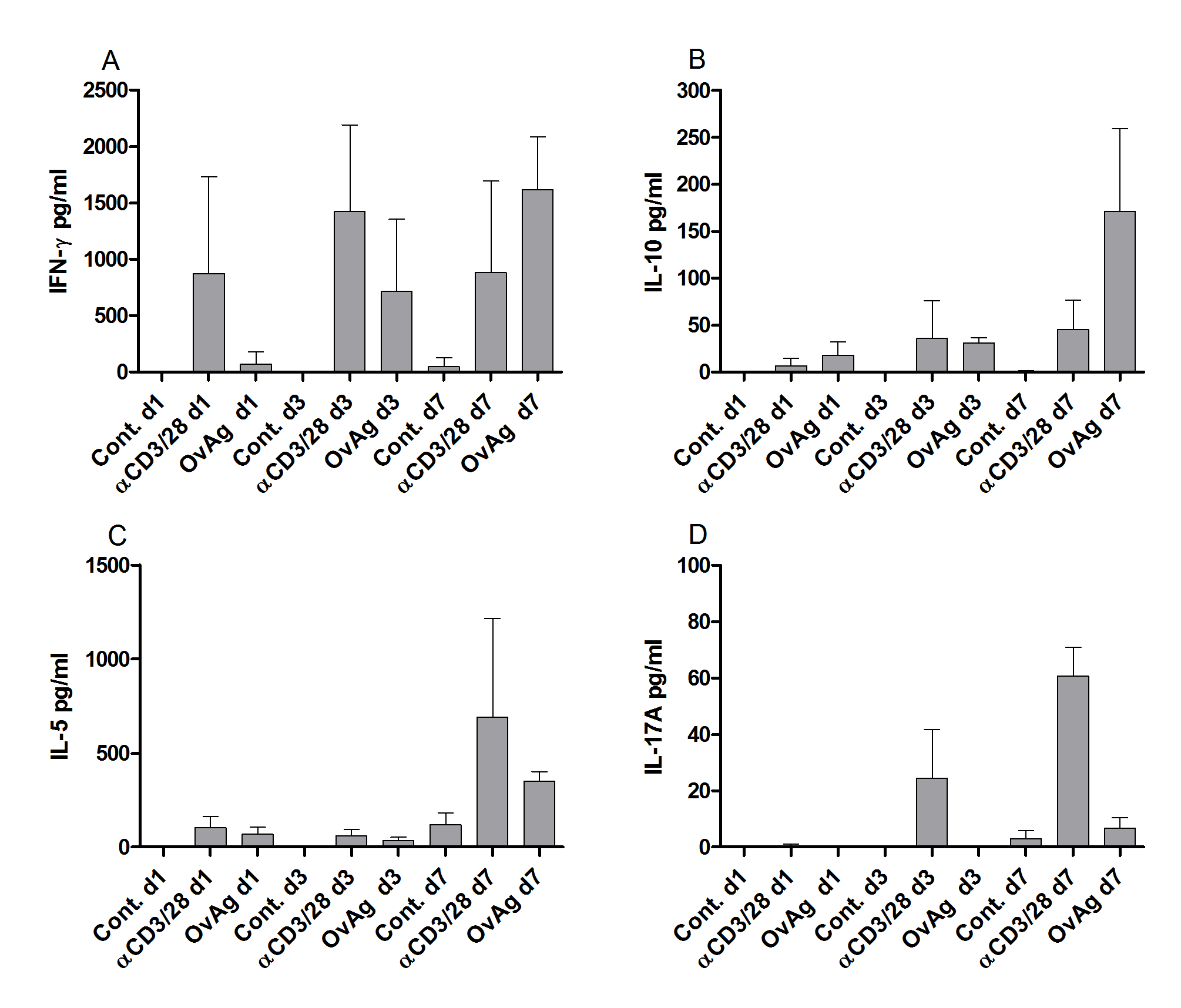

Supplement: S1 Fig — Optimal time point for collecting cell culture supernatants. Thawed PBMCs (1×10^5^/well) from Onchocerca volvulus-infection free individuals (n = 4) were left alone (Cont.) or stimulated with either O. volvulus antigen extract (20 µg/ml) or αCD3/αCD28 (40,000 beads/ml). Supernatants were collected on day 1 (d1), 3 (d3) and 7(d7). Secretion levels of IFN-γ (A), IL-10 (B), IL-5 (C), and IL-17A (D) were then measured by ELISA. Bars represent mean ± SD of cytokines levels. (TIF) [file pntd.0003414.s001.tif]
